# Fake gunshot wounds in the skull—post-mortem artifact caused by steel probe during police search for a missing body

**DOI:** 10.1007/s00414-020-02420-y

**Published:** 2020-09-09

**Authors:** Michał Kaliszan, Wojciech Dalewski, Joanna Dawidowska, Tomasz Gos, Zbigniew Jankowski

**Affiliations:** Department of Forensic Medicine, Medical University of Gdańsk, ul. Dębowa 23, 80-204 Gdańsk, Poland

**Keywords:** Forensic pathology, Post-mortem artifact, Steel probe, Head injury, Homicide, Gunshot wound

## Abstract

The paper presents a case of a forensic autopsy of a young woman who was murdered and her dismembered body was hidden in soil and water. In the skull of the deceased, in the temporal and occipital regions, the autopsy revealed 3 round, almost identical holes, which looked like small caliber gunshot wounds. Doubts about the cause of these injuries were raised by the fact that despite the decomposition of the body, the continuity of the dura at the site of these holes remained undamaged and the absence of any trace of a bullet’s wound track in the brain, the absence of a foreign body in the cranial cavity, as well as the absence of wounds on the opposite side of the skull that could be exit wounds. A thorough analysis of the investigation and the activities carried out during the search for the missing body allowed to adopt and finally confirm the hypothesis that the above mentioned skull damage occurred during the search for the cut-off head of the deceased in shallow water by means of special tapered conical steel probes used by the rescue/search teams. Due to the structure of such a spike, i.e., a sharp end and then a wide cone, only a superficial puncture of the steel probe tip three times into the skull had taken place, which caused regular, rounded bone damage without damaging the dura and brain. The presented case indicates that sometimes post-mortem artifacts may suggest a completely different origin of wounds, which emphasizes the need for a comprehensive analysis of all possible causes of their occurrence, particularly data concerning the handling of the corpse before it is delivered to the morgue, so as not to make a diagnostic error during autopsy.

## Introduction

In the case of hiding a corpse and the longer time that elapsed from death to body discovery, one should take into account the possibility of post-mortem injuries caused both by environmental conditions (taphonomic changes), by the action of living organisms (insects and their larvae or other animals), as well as by the actions of humans during the search or recovery of the corpse [1–4]. The distinction between vital injuries and post-mortem artifacts may sometimes be a great challenge for the forensic pathologist performing the post-mortem examination [5]. So far, the literature has described various interesting cases of post-mortem injuries differentiated from vital ones, mainly caused by animals [6–12]. However, no case description was found in available literature, as described below.

## Case report

A young woman, who went missing a month before, was murdered by her boyfriend, who then decided to hide the body and complicate its identification, if found. For this purpose, he dismembered the body by cutting off the head, torso and limbs, and removing large areas of skin from the body parts, including the face and fingertips. He buried fragments of her body separately in different places and sunk the skinless head in a shallow water reservoir with a very muddy bottom. When, after family had reported the woman missing, the police began searching for her and found traces indicating that the man may have murdered his partner, he confessed to the murder and indicated where the body fragments were hidden. The murder was supposed to have happened about 1 month earlier. The body and limb fragments were dug out quickly and then subjected to an autopsy. However, the search for the missing head continued, due to the sinking of the head in water. After 2 days, the head, with part of the neck, was found and also sent for post-mortem examination. The head was key in determining the cause of death, as it revealed injuries, including a fracture of the mandible and damage of the hyoid bone, which indicated manual strangulation as the cause of death and correlated with the suspect's testimony.

**Fig. 1:**
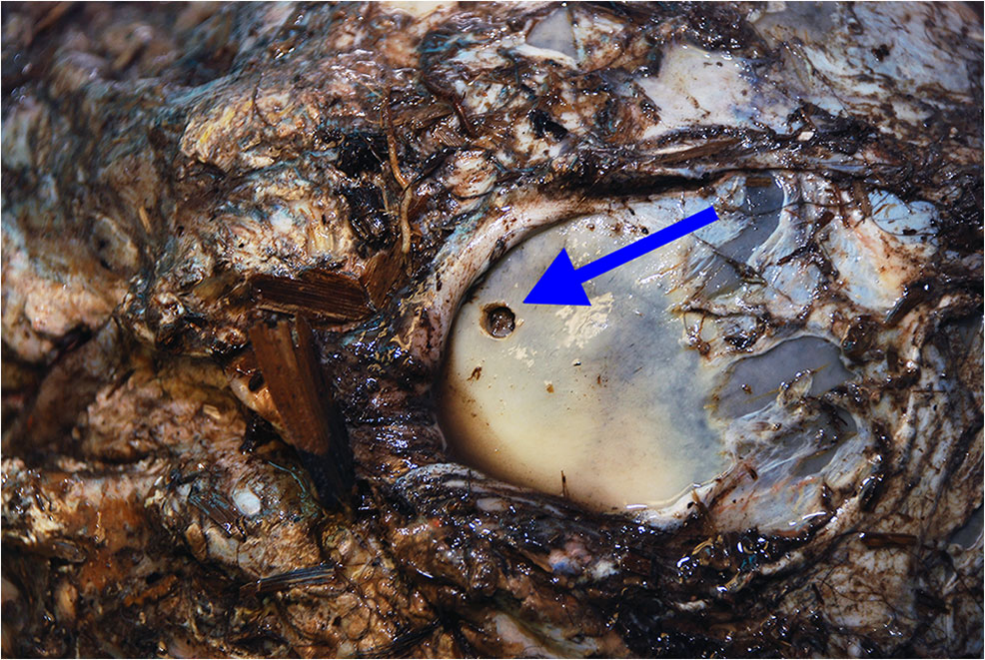
Round hole in the temporal part of the skull (arrow) visible after soft tissue removal

**Fig. 2:**
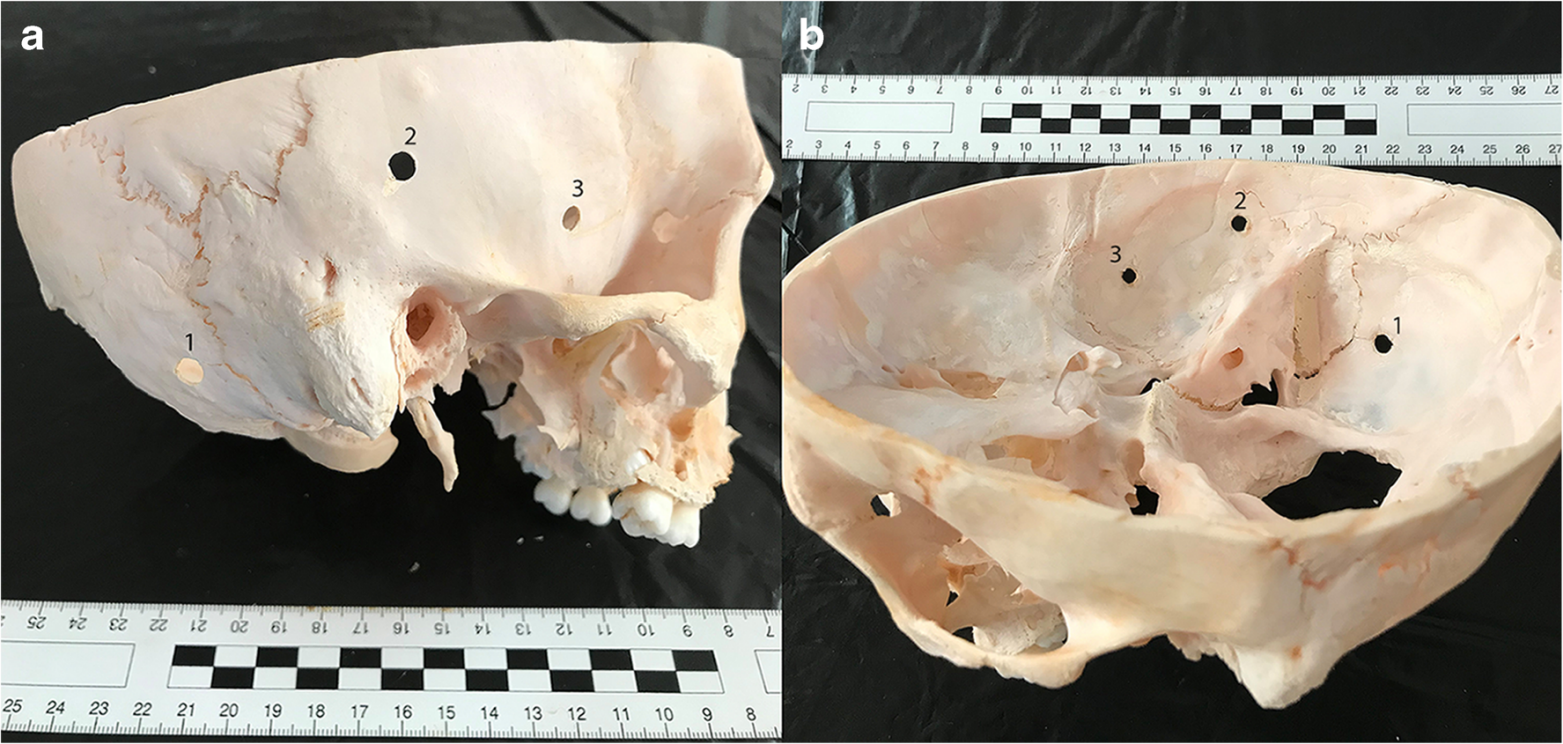
Three round holes (1, 2, 3) visible in the macerated skull bone, from the outside (**a**) and inside (**b**)

**Fig. 3:**
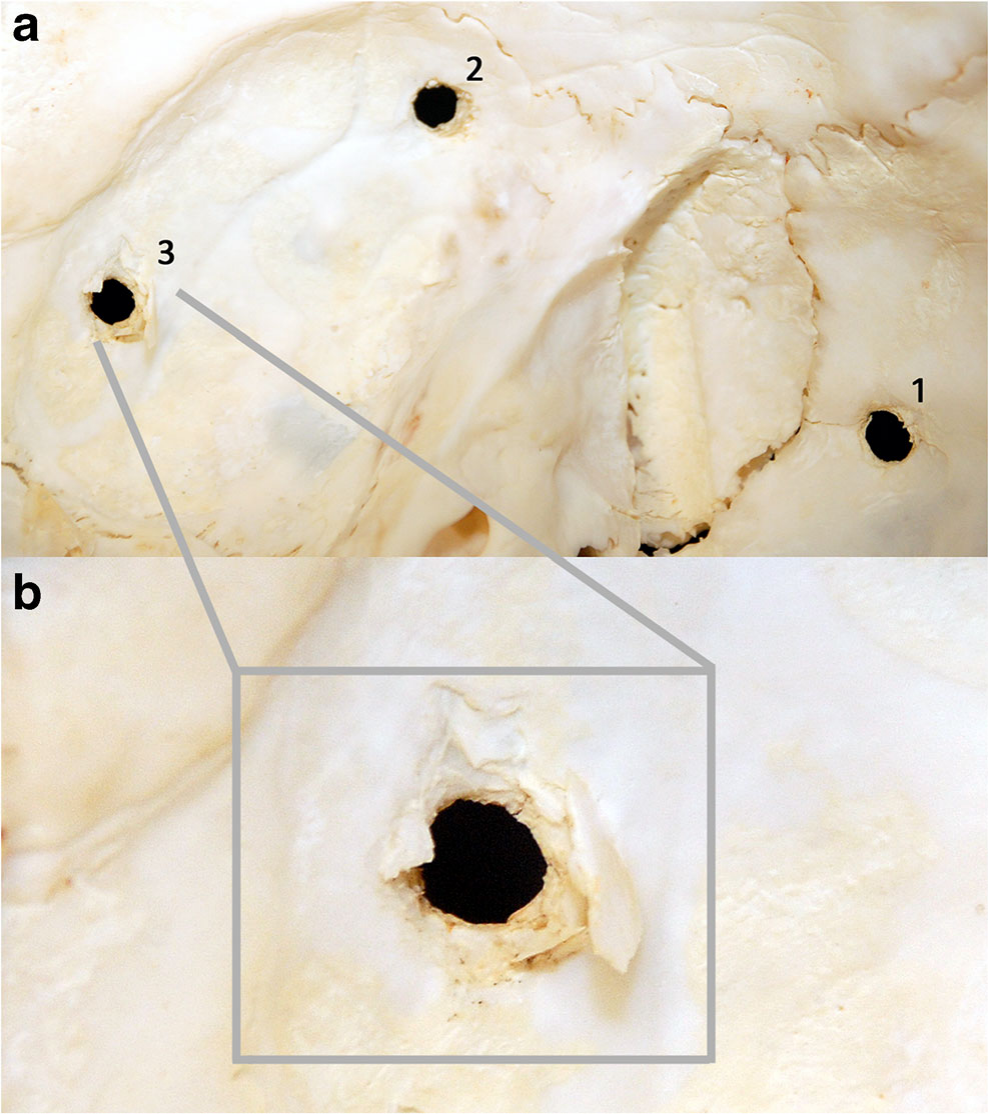
Close up of the holes (from the inside) with clearly visible slightly recessed bone fragments in its margins and funnel-shaped inward extensions

During the post-mortem examination of the head, after separation of the preserved soft tissues exhibiting decayed lesions, apart from the above mentioned mandibular injuries, an atypical finding was revealed in the form of three round holes, each 0.5 cm in diameter, with smooth external edges located in the right temporal bone and slightly backwards in the occipital bone (Fig. 1). At first glance, the holes resembled gunshot wounds from a small caliber weapon. However, there was no damage to the dura mater, which was flaccid, separated from the inner surface of the skull, but had a preserved continuity. No damage to the brain structure or the presence of foreign bodies inside the skull, such as bullet traces, was found. Due to the unusual finding in the skull, it was macerated and then reassessed (Fig. 2). The holes in the bones were almost identical, located a few centimeters from each other. During detailed examination of the macerated skull bone, the presence of a funnel-shaped inward extension in the bone in the place of the holes was noted what is common for entrance gunshot wounds. Also, small slightly recessed bone fragments were visible on the inside of the skull in the holes margins. There were no fractures in the skull presenting as fissures diverging radially from the holes in the bone (Fig. 3).

## Discussion

In light of the above, several hypotheses appeared regarding the time of occurrence and cause of the mysterious 3 holes in the skull. Due to the lack of damage to the dura mater and brain, with the present damage covering the entire thickness of the skull (0.3 cm), the origin of bone damage from a shot from a firearm or pneumatic weapon was excluded. It is difficult to imagine a situation in which a bullet damages the entire thickness of the skull but stops on the dura mater and does not enter the cranial cavity [13–15]. In the case of gunshot wounds, besides presence of a funnel-shaped inward extension in the bone, often radial fracture fissures appear around the wound in the bone [13, 14]. Such fissures in our case were not present. Only a small, linear bone fracture was found on the edge of one of the holes (Fig. 3—hole no. 1 visible from the inside).

**Fig. 4:**
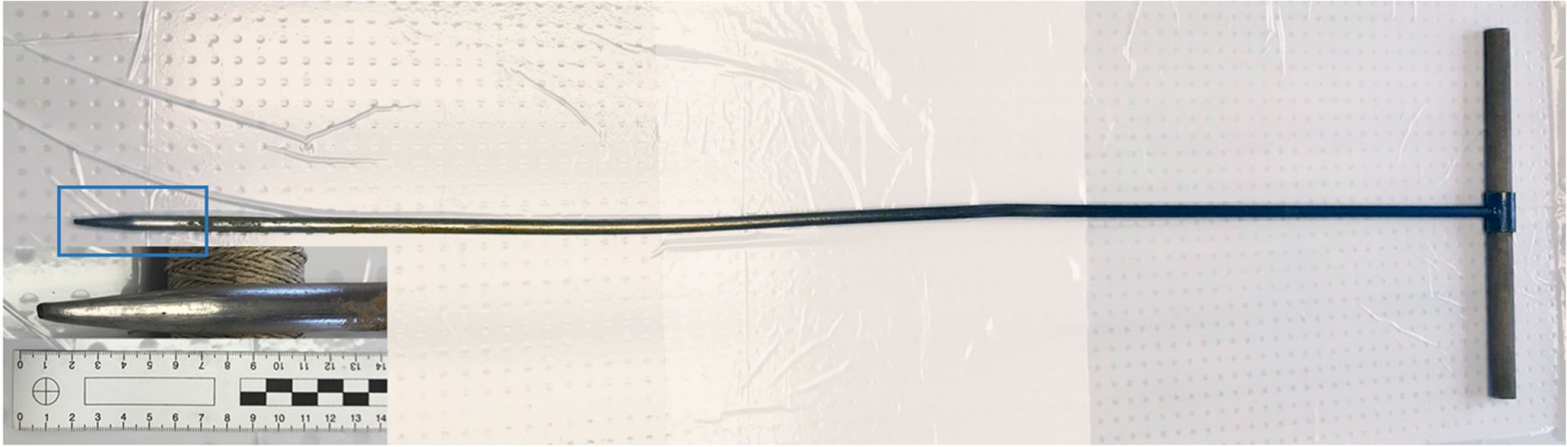
The steel probe used for searching for the buried body, its ending zoomed in the corner

**Fig. 5:**
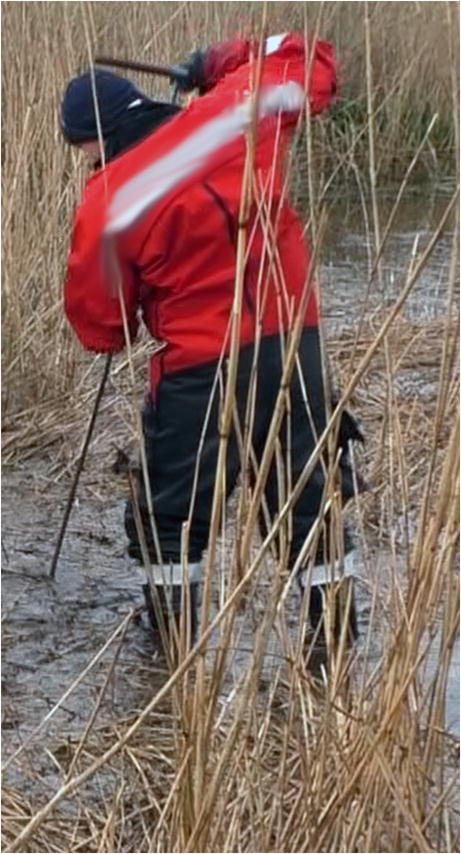
Search for a body with use of steel probe (illustrative image)

**Fig. 6:**
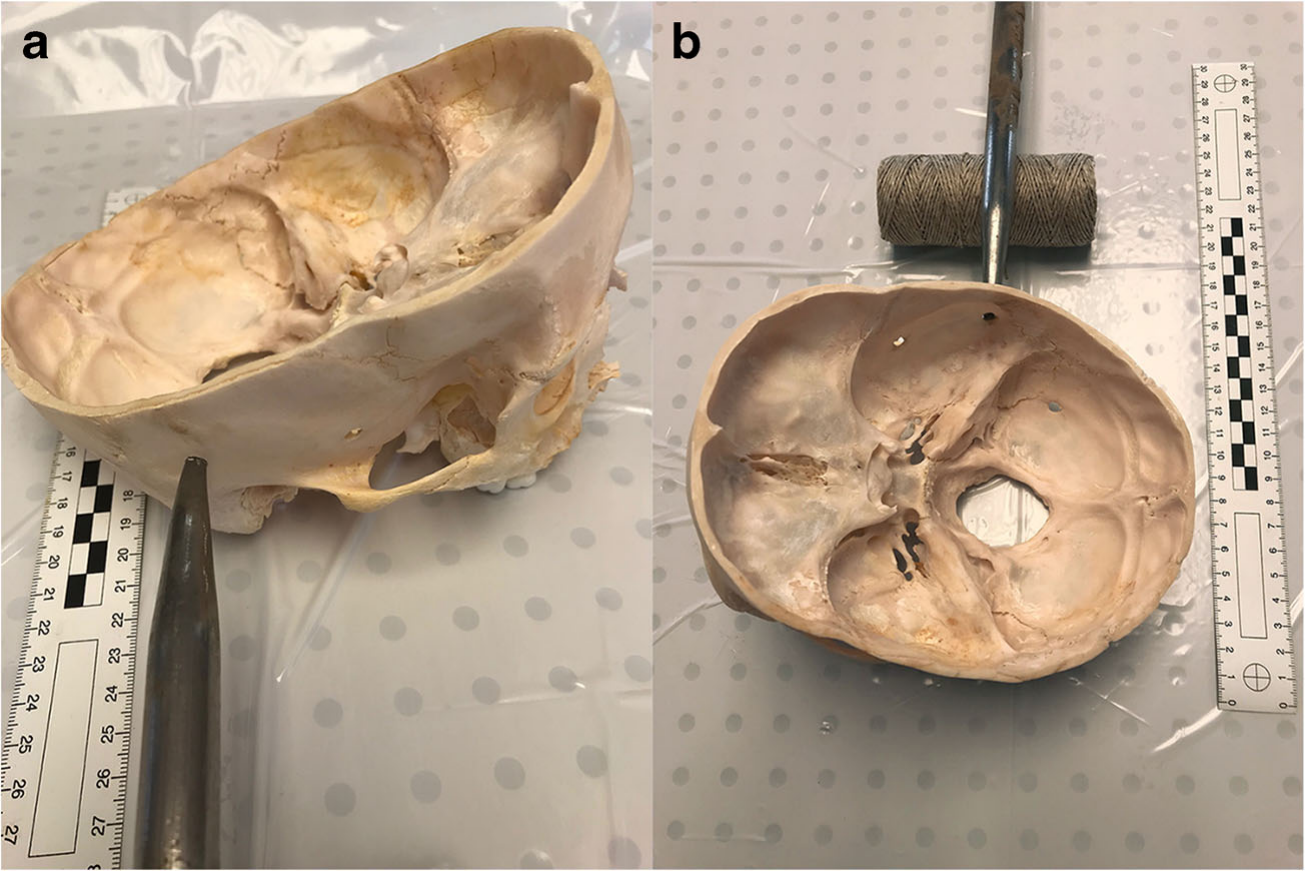
Ending of the steel probe accurately fitting the round hole in the skull, visible from the outside (**a**) and inside (**b**)

The remaining hypotheses concerned other possibilities of skull bone injuries. One of them was the possibility of a neurosurgical procedure in the past despite the uncharacteristic hole size for such procedures [16]. Nevertheless, this hypothesis was excluded by the family of the deceased. Another hypothesis was that the suspect could have damaged the skull with a drill, either while the victim was still alive, or after her death [17, 18]. He did not confess to such deeds, no drill was found, and it would have been very unlikely that he would have drilled such thin bone completely three times without damaging the dura mater and brain in any of them. So this version also remained very doubtful. When speculating with the policemen about the possible causes of such puzzling holes, a question arose about the search for the head in a muddy water reservoir and how it was eventually discovered. It turned out, that during the search for buried corpses, special steel probes are used (similarly to the search for victims of an avalanche), which are punched into the ground [19]. These probes have a twofold use: they create a thin channel in the ground so that police dogs can smell a corpse, or they can be used to find a corpse when the probe end hits the corpse directly and stops at it (Fig. 4). People who took part in the search for the head using steel probes were interviewed and one of them confirmed that during the punching of the probe into the bottom of the water reservoir, shortly before the head was found, he felt resistance. Furthermore, a recording of the search was obtained on which the manner of searching for the head in the water reservoir with the use of a steel probe had been recorded (Fig. 5). After this information was provided, the steel probe was brought to the morgue and its structure was compared with the holes in the skull, which allowed the mysterious holes’ puzzle to be solved. Therefore, taking into account the above, it can be assumed that during the search for a body using the above mentioned steel probes, the right side of the skull, which was hidden in a silt in a shallow water, was punctured three times before it was recovered. Due to the characteristic structure of the steel probe, i.e., sharp but short ending, with a significantly widening conical shaft, no deeper puncture was caused to the skull. The only damage caused by the sharp ending of the probe was the round hole in the 0.3-cm thick skull bone (Fig. 6).

## Conclusions

This case shows how important and helpful is the cooperation between forensic pathologists performing post-mortem examinations and law enforcement and judicial investigators at every stage of an investigation. Thanks to a joint effort, it was possible to find the cause of initially very unusual looking skull injuries in a murder case, and establish that it was a post-mortem artifact, not inflicted by the perpetrator. The presented case was facilitated because the head was found about 1 month after the murder, when soft tissues, including the dura mater and the brain were still preserved. The post-mortem examination would have been much more difficult if during the search, despite causing the head punctures, the head would not have been found at the time of investigation, but e.g. several months or years later when there would be no more soft tissue, only bone. Then, one could come to the wrong conclusion that the cause of death were gunshot wounds to the head, and the bullets or their fragments were washed out by water or were lost while recovering the skull from the water. This case also highlights the variety of post-mortem injuries and draws attention to the necessary vigilance when diagnosing injuries, especially on a body which was found some time after death.
